# Awareness of Eating Disorders, Nutritional Knowledge, and Emotionally Driven Eating Among Polish Adolescents Aged 15–17—A Pilot Study

**DOI:** 10.3390/nu17121994

**Published:** 2025-06-13

**Authors:** Marlena Zięba, Marta Jaskuła, Sabina Lachowicz-Wiśniewska

**Affiliations:** Faculty of Medicine and Health Science, University of Kalisz, Wojciecha Bogusławskiego Square 2, 62-800 Kalisz, Poland; marlenaannazieba@gmail.com (M.Z.); m.jaskula@uniwersytetkaliski.edu.pl (M.J.)

**Keywords:** eating disorders, adolescent awareness, prevalence of disorders

## Abstract

**Background/Objectives:** Despite the growing awareness of nutrition and the popularity of healthy lifestyles among adolescents, disordered eating behaviors—such as anorexia nervosa, bulimia nervosa, binge eating disorder (BED), and avoidant/restrictive food intake disorder (ARFID)—remain significant public health concerns. ARFID, officially recognized only in 2013, is still poorly understood among youth. This study aimed to assess the relationship between adolescents’ nutritional knowledge, emotional regulation, media influence, and eating behaviors. **Methods:** A cross-sectional study was conducted in 2024 among 120 students aged 15–17 attending W. Reymont Secondary School No. II in Ostrów Wielkopolski, Poland. Participants completed a custom-designed, paper-based questionnaire consisting of 30 single-choice questions and demographic items. The instrument assessed knowledge of eating disorders, body satisfaction, social media impact, and the emotional determinants of food choices. The tool was developed with expert input but has not undergone formal psychometric validation. While many adolescents demonstrated basic nutritional knowledge—such as awareness of BMI norms and food group distribution—they often failed to apply this knowledge to their dietary behaviors. **Results:** Significant gender differences were observed: girls were more likely to restrict food intake, report emotional eating, and engage in slimming behaviors, while boys were less emotionally reactive and less influenced by social media. Most participants reported eating one meal daily with family but rarely discussed nutrition at home. Emotional involvement in eating, particularly among girls, emerged as a key factor, more influential than social media in shaping dietary behaviors. **Conclusions:** The findings highlight a clear gap between nutritional knowledge and actual behavior among adolescents, driven in part by emotional dysregulation and body image concerns. School-based interventions should incorporate not only nutritional education but also emotional regulation strategies and media literacy to effectively support healthy eating behaviors in youth.

## 1. Introduction

Healthy nutrition during adolescence and early adulthood is a fundamental determinant of long-term physical and mental well-being. This developmental phase, encompassing individuals aged 15 to 19, is marked by intense physiological, psychological, and social changes that significantly shape dietary behaviors and health outcomes in adulthood. Dietary habits formed during childhood, often influenced by parental modeling, undergo substantial transformations in adolescence due to peer influence, media exposure, and growing societal pressure concerning body image [1,2,3,4,5,6,7].

Yet despite increased exposure to health messaging, adolescents frequently struggle to apply nutritional principles in practice. Studies indicate that many young people regularly skip meals, consume nutritionally unbalanced foods, or engage in restrictive dieting, particularly girls. This gap between theoretical understanding and practical application often results in dietary deficiencies, poor metabolic outcomes, and the development of disordered eating behaviors [6,7,8]. Nutritional awareness can be defined as an individual’s declarative knowledge regarding dietary guidelines, including awareness of food group distribution, caloric content, BMI interpretation, and health-related nutritional recommendations [5,7,8]. However, such knowledge does not guarantee healthy eating behaviors, as this depends on one’s emotional regulation capacity, environmental factors, and internal motivation.

Disorders such as anorexia nervosa (AN), bulimia nervosa (BN), binge eating disorder (BED), and avoidant/restrictive food intake disorder (ARFID) have become increasingly prevalent among adolescents. According to current epidemiological data, approximately 40% of young women report symptoms of anorexia, 1.5% of bulimia, and 3.5% of BED, while among young men the prevalence is lower (0.1%, 0.7%, and 2%, respectively). These disorders are multifactorial in nature, involving emotional, familial, cultural, and environmental determinants. ARFID, officially classified as a clinical condition only in 2013 by the DSM-5, is particularly complex, as it stems not from body image dissatisfaction but from strong aversions to food texture, color, or traumatic associations with eating [9,10,11]. A growing body of research indicates that emotionally driven eating—the consumption of food in response to negative affective states such as anxiety, boredom, or sadness—is a critical behavioral pattern among adolescents [8]. This form of eating is thought to reflect underlying deficits in emotional regulation, a psychological process that enables individuals to monitor, evaluate, and modify emotional reactions in adaptive ways. During adolescence, emotional regulation circuits—especially those governed by the prefrontal cortex—are still maturing, which increases vulnerability to emotionally mediated maladaptive behaviors, including disordered eating [8].

Moreover, the phenomenon of anorectic readiness—characterized by excessive control over food intake, weight preoccupation, and perfectionism—has been identified as a preclinical syndrome that often precedes the manifestation of full-blown eating disorders [2,12]. These behaviors are often reinforced by poor emotional regulation, especially during puberty, when adolescents are particularly vulnerable to anxiety, depression, and peer pressure.

Importantly, the role of media, especially social media, cannot be overlooked. Platforms like Instagram or TikTok perpetuate unrealistic standards of beauty and body image, often leading adolescents to adopt unsafe eating behaviors [13]. Szałachowski and Tuszyńska-Bogucka [13] show that social media exposure leads to increased use of restrictive diets and preoccupation with calorie content. Similarly, other authors observed that social media exposure correlated with obsessive behaviors concerning calorie counting and emotional eating [14,15,16,17].

In light of these challenges, nutrition education emerges as a pivotal strategy for promoting health and preventing the onset of eating disorders. Schools are particularly effective settings for such interventions, given their access to adolescents at a formative stage of behavioral development. Education should not be limited to dietary recommendations but should include psychological components such as body image resilience, media literacy, and strategies to manage emotional triggers related to food. Evidence suggests that adolescents who participate in structured nutrition education programs are more likely to make healthier dietary choices and avoid ultra-processed foods [12,17,18].

However, the absence of reliable, consistent education on these topics may contribute to adverse health outcomes, including malnutrition, overweight, or obesity—all of which elevate the risk of metabolic syndrome, cardiovascular disease, and emotional disturbances. Early education significantly lowers the incidence of diet-related illnesses later in life [17,19].

Despite the importance of these findings, several research gaps persist. Core constructs such as nutritional awareness and emotionally driven eating are rarely operationalized in adolescent populations, making cross-study comparisons difficult. Furthermore, existing studies seldom incorporate affective or behavioral regulation theories (e.g., dual-process models or coping frameworks) that could explain the discrepancy between knowledge and action. For instance, emotion dysregulation has been identified as a transdiagnostic mechanism linking psychopathological traits and maladaptive eating [8,10,19].

Given this context, the current study aimed to assess the level of nutritional awareness, the prevalence of emotionally driven eating, and the influence of media and familial context on dietary behaviors in adolescents aged 15–17. We hypothesized that (i) girls would report higher emotional involvement in eating behaviors and greater susceptibility to media influence; (ii) nutritional awareness would not consistently predict healthy dietary habits; and (iii) emotionally driven eating would be associated with body dissatisfaction and restrictive dieting. This work contributes to ongoing discourse by integrating behavioral and emotional dimensions of adolescent eating patterns and by situating them within the psychosocial landscape of Polish youth. Ultimately, findings may help inform preventive strategies that target both cognitive and emotional pathways to disordered eating.

## 2. Materials and Methods

### 2.1. Study Design and Participants

This cross-sectional study was conducted in 2024 among students aged 15–17 attending the Władysław Reymont Secondary School No. II, a public school located in a medium-sized city in central Poland. The study aimed to assess the level of nutritional awareness and knowledge related to eating disorders among adolescents aged 15 to 17 years. A total of 120 participants were recruited, all of whom were full-time students at the selected high school. The inclusion criteria consisted of age (15–17 years), school enrollment at the time of the study, and provision of informed consent. The age range of 15–17 years was selected due to logistical access and institutional approvals within that educational setting, although we acknowledge this does not fully represent the broader age range implied by the study title (15–17 years). Prior to participation, the aim and scope of the study were clearly explained to the students, and written informed consent was obtained. For participants under the age of 18, parental or legal guardian consent was also secured. All participation was voluntary, and confidentiality was assured. The research protocol was approved by the Committee for Scientific Research Ethics of the University of Kalisz (Approval No. KB-551/2022, date: 29 April 2024), and the study was carried out in accordance with the Declaration of Helsinki [20,21,22].

### 2.2. Research Tools and Data Collection

The study employed a diagnostic survey method using a custom-designed questionnaire developed specifically for this research. The questionnaire was administered in a paper-based, in-person format and was completed anonymously during supervised sessions in the school setting. The questionnaire consisted of 30 single-choice, open-ended questions, along with a demographic section. The items addressed multiple aspects, including the following (Table S1):

(a) Knowledge and awareness of disordered eating behaviors: anorexia nervosa (AN), bulimia nervosa (BN), binge eating disorder (BED), and avoidant/restrictive food intake disorder (ARFID).
(b) Attitudes toward body image and body satisfaction.
(c) Perceptions of healthy eating and understanding of concepts such as BMI and the Healthy Eating Plate.
(d) The influence of emotions on food choices and the perceived impact of family, peers, and media.
(e) Awareness of the importance of regular meal consumption and healthy food intake.

The demographic data collected included age, gender, place of residence, and self-reported body mass index (BMI). Lifestyle-related questions addressed physical activity levels, use of stimulants (alcohol, cigarettes), frequency of meals, and patterns of food intake, including snack consumption and communal eating. The questionnaire was reviewed by two independent experts in adolescent psychology and nutritional sciences to assess clarity, content relevance, and age-appropriate comprehension. The questions were formulated to evaluate both declarative knowledge and personal beliefs. The collection of responses was conducted anonymously to ensure honesty and reduce response bias. The questionnaire used in this study was developed by the authors based on a review of the existing literature on adolescent nutrition and eating behaviors. However, the instrument has not undergone formal psychometric validation or pilot testing. The instrument has not undergone formal psychometric validation or pilot testing; therefore, its results should be interpreted with caution and considered exploratory in nature. Moreover, the questionnaire did not collect information on participants’ socioeconomic status (SES), mental health diagnoses, or prior psychological treatment, limiting the ability to control for potential confounding variables in the statistical analysis. These omissions represent important methodological limitations of the present study.

### 2.3. Statistical Analysis

All collected data were systematically coded and entered into a spreadsheet for analysis. Quantitative analyses were carried out using Microsoft Excel and STATISTICA software (version 13.3 PL; StatSoft Inc., Tulsa, OK, USA; StatSoft, Kraków, Poland). Descriptive statistics (frequencies and percentages) were used to summarize the data. Chi-square tests for independence and Pearson’s correlation coefficients were applied to assess associations between categorical and continuous variables, respectively. Due to the exploratory nature of this pilot study and the modest sample size, analyses were not adjusted for multiple comparisons or for potential confounding variables such as SES or BMI. Thus, statistical findings should be interpreted with caution. No power calculation was performed a priori, as the study was intended as a hypothesis-generating investigation. The threshold for statistical significance was set at *p* < 0.05, together with the correlation coefficient (Cramer’s V) (Table S2). Data were visually inspected using bar graphs and pie charts to enhance interpretability where applicable. In addition to descriptive and bivariate analyses, we constructed several composite indices to quantify behavioral and psychosocial patterns related to nutrition. The Emotional Eating Index (EEI) was calculated based on four survey items covering eating under stress, sadness, guilt, and episodes of overeating. Each item was scored from 0 to 2, yielding a total EEI score from 0 to 8. The Nutrition Knowledge Index was derived from four factual questions regarding healthy eating guidelines, BMI ranges, satiety, and eating disorder awareness (each scored 0 or 1). A behavior score was calculated from three indicators: regular breakfast consumption, vegetable intake, and physical activity (0–3 points). The knowledge–behavior gap was computed as the difference between the knowledge and behavior scores. Additionally, the Media and Body Pressure Index was computed using items related to social media influence and body comparison. Gender-based comparisons of these indices were summarized in Supplementary Table S4. Categorical variables were also cross tabulated by gender and assessed using chi-square tests of independence, with results presented in Supplementary Table S3. For descriptive purposes, participants were categorized into low, moderate, and high levels for both knowledge and knowledge–behavior gap using predefined scoring thresholds. These distributions were summarized in Supplementary Table S5.

## 3. Results

The survey conducted targeted adolescents. Among the respondents, females constituted the majority, accounting for 70%, with the highest representation among 16- and 17-year-olds (21% and 42%, respectively). Male participants represented only 30% of the sample, with 16-year-olds (47%) and 17-year-olds (38%) being the most prominent age groups. The majority of surveyed females resided in small towns (66%), followed by medium-sized towns (20%), while significantly fewer lived in rural areas (13%) or large cities (1%). In contrast, young males most frequently reported living in large cities, followed by medium-sized towns, with small towns and rural areas being the least represented (9%). The average BMI among most participants fell within the normal range. The remaining respondents were classified as underweight, with a BMI below 18.5 (Table 1).

### 3.1. Healthy Eating and Lifestyle as a Value for Adolescents

**Figure 1:**
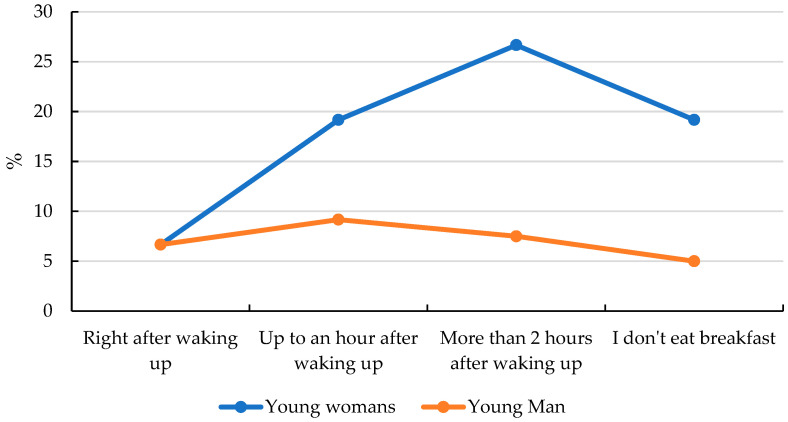
Declared breakfast time for young women and young men.

The first question addressed to the respondents was the following: *“How soon after waking up do you eat your first meal?”* The majority of adolescents (34%) reported eating breakfast more than two hours after waking up—suggesting that their first meal may be consumed at school. Slightly fewer respondents (28%) indicated eating breakfast within one hour of waking, while a comparable portion (24%) stated that they skipped breakfast altogether. Only 14% reported eating breakfast immediately after waking (Figure 1 and Table S1). The results that were obtained were statistically significant, and the correlation coefficient was 0.270 (Table S2). These responses highlight the potential need to implement educational programs in schools to raise awareness about the importance of consuming the first meal of the day.

In response to the question “*Is regular meal consumption important to you?*” the majority of adolescents (76%) indicated that regular meals are not important to them (Figure S1). This perception may be associated with a lack of parental guidance regarding healthy eating. This interpretation is supported by responses to the question, “*Do you talk with your parents/guardians at home about healthy eating?*”—with 68% of adolescents stating that such conversations do not take place at home. Furthermore, when asked, “*Do you eat at least one meal a day with your family?*” the answers were divided: only 56% reported sharing at least one meal per day with their family, and 24% indicated they do so only on weekends. Alarmingly, 13% of respondents declared that they do not eat with their parents at all (Figure S2). These findings clearly emphasize the importance of implementing nutrition education among adolescents. However, they also suggest the potential value of extending educational efforts to parents to raise awareness about the importance of proper nutrition and its impact on child development and long-term health.

### 3.2. Adolescents’ Eating Behaviors and Their Attitudes Toward Nutrition

The next section of the survey focused on the daily eating behaviors and lifestyle habits of adolescents. In response to the question *“Do you ever deliberately skip a meal?”*, the majority of respondents stated that they do not intentionally skip meals (41%), while 32% indicated they do so occasionally, and 27% admitted to deliberately skipping meals. Gender differences were observed, with adolescent girls more frequently reporting intentional meal skipping (28%) compared to boys (5%). Indeed, most men do not skip meals on purpose, whereas most women do skip them, with the correlation coefficient being 0.234 (Table S2). Skipping meals may be associated with various factors, such as attempts to reduce body weight or poor time management that interferes with regular eating patterns (Table S1).

**Figure 2:**
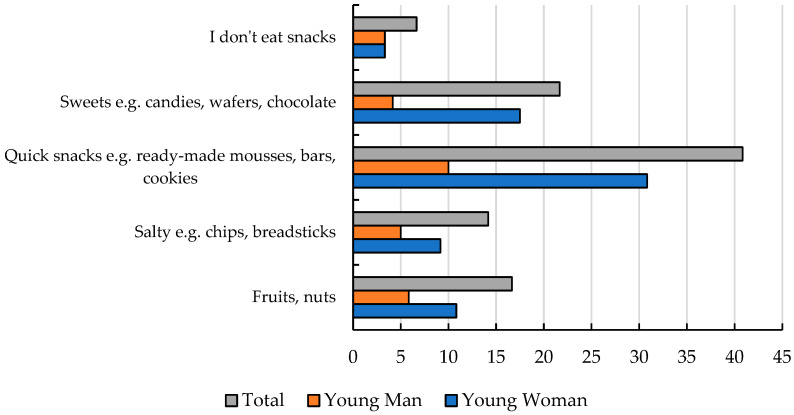
Declared consumption of snacks [%].

**Figure 3:**
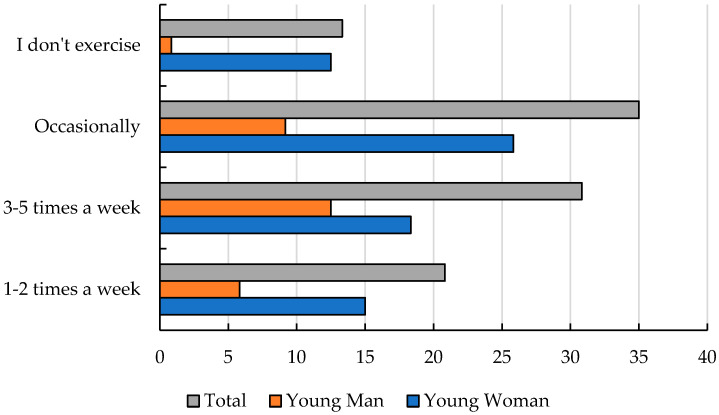
Declaring the frequency of practicing sports outside of school [%].

When asked, “Do you add a serving of vegetables to your main meals?” most adolescents responded that they sometimes add vegetables (64%), followed by always (28%), and do not like vegetables (8%). Girls reported skipping meals more frequently than boys, although this was not controlled for BMI, parental influence, or exposure to dieting content (22% vs. 7%, respectively) (Table S1). These findings indicate a general deficiency in vegetable consumption among adolescents, which may result in insufficient intake of dietary fiber and folic acid. Adolescents also showed a low tendency to choose healthy snacks between meals. This dietary behavior may exacerbate nutritional deficiencies and reflect a poor overall attitude toward healthy eating. This is supported by responses to the question *“What types of snacks do you consume during the day?”*, where the most frequently consumed snacks were fast snacks (41%) and sweets (22%), followed by fruits and nuts (17%), salty snacks (14%), and no snacks (6%) (Figure 2). A promising result emerged from the responses to the question *“Do you use any substances*, *such as alcohol*, *cigarettes*, *or others?”*—with the majority of adolescents indicating no substance use (68%), 25% reporting occasional use, and only 7% using them frequently (including 5% of girls and 3% of boys) (Table S1). In addition to mandatory physical education classes, adolescents reported engaging in physical activity during the week. In response to the question *“How often do you engage in physical activity?”*, a significant majority (87%) indicated that they incorporate some form of physical activity into their routines. Most adolescents (61%) reported that they do not experience stress when eating in the presence of others. However, 33% indicated that they feel stress when eating with peers, and 6% when eating with family members (Figure 3).

### 3.3. Emotionally Driven Eating and Disordered Behaviors Among Adolescents

Although emotional eating was not assessed using a standardized or validated scale such as the Emotional Eating Questionnaire (EEQ), the survey included multiple items addressing behaviors typically associated with emotional eating, such as eating in response to stress, boredom, or sadness. Therefore, the results reported here should be interpreted as indicative rather than diagnostic. These responses may reflect a limited engagement with weight monitoring; however, no validated tools were used to assess this construct. Female participants more frequently reported comparing their appearance with others. This finding was statistically significant but not adjusted for potential mediators such as BMI or media usage.

Although adolescents were asked, “Do you pay attention to your daily calorie intake?” the majority (63%) responded that they do not track calories, while 29% estimate their intake “by eye”, and only 8% reported using mobile applications to monitor their consumption. Women are significantly more likely to avoid products due to the risk of gaining weight, while almost half of men do not think about it at all, with the correlation coefficient being at the level of 0.266 (Table S2). These self-reported responses may reflect a limited engagement with body weight monitoring; however, without validated tools or longitudinal data, this conclusion should be interpreted cautiously. This is further supported by responses to the question, *“How often do you check your body weight?”*. The largest group (33%) stated that they weigh themselves only once a year, while 25% reported doing so either monthly or weekly, and only 10% indicated that they either do not monitor their weight at all or do so daily (Table S1).

**Figure 4:**
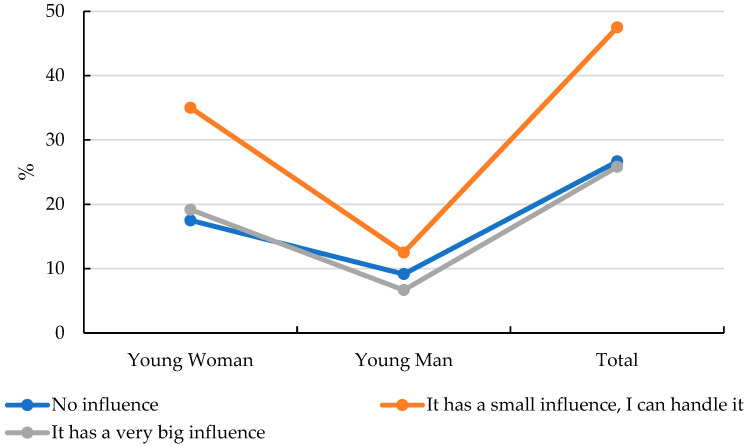
Respondents’ declaration on the influence of social media on the perception of their appearance.

When asked, “Do you avoid specific food products out of concern that they might cause weight gain?” opinions were divided. The largest group (38%) claimed they do not consider whether certain foods could lead to weight gain. A similar proportion (33%) stated that they consume such products in moderation, while 29% acknowledged consciously thinking about the potential for weight gain from certain foods. These behaviors may be partially influenced by exposure to idealized body images and peer comparison, especially on social media platforms. Women were significantly more likely to undergo weight loss treatments than men, with the correlation coefficient being 0.322 (Table S2). In response to the question *“Do you compare your body to others?”*, the majority (74%) stated that they sometimes or frequently make such comparisons. This finding was statistically significant but not adjusted for potential mediators such as BMI or social media use frequency. With the correlation coefficient being 0.266 (Table S2). Similarly, when asked, *“To what extent do social media influence how you perceive your appearance?”*, 74% acknowledged that social media affects their body image perception, although 47% of them reported being able to cope with this influence (Figure 4).

Adolescence is a period often accompanied by intense stress and a variety of developmental challenges, including body image concerns and emotional sensitivity to everyday situations. Questions related to emotional eating under stress reveal similar behavior patterns among adolescents. In response to the question, *“Do you tend to reach for fast food when experiencing intense stress?”*, 32% of respondents reported that they either “rather do” or “always do” turn to fast food during such moments. Conversely, 69% indicated that they “rather do not” or “never” consume fast food in response to stress. Gender differences were evident: young women were more likely (24%) than young men (8%) to reach for such foods during stressful periods (Table S1). Regarding the question, *“Do you experience episodes of overeating when feeling sad*, *doubtful*, *bored*, *or under significant stress?”*, 39% of respondents stated that this occurs occasionally, and 15% reported that it happens every time they experience such emotions. The remaining 46% claimed not to experience binge eating episodes. A gender breakdown revealed that 49% of young women reported such experiences, compared to only 6% of young men (Table S1). Indeed, the vast majority of men do not experience binge eating, while almost half of women do, with the correlation coefficient being at the level of 0.428 (Table S2).

When asked, *“Do you feel guilty after consuming something unhealthy or in excessive amounts?”* responses were equally divided: 50% reported not feeling guilty, while 37% said they sometimes do, and 13% admitted they always experience guilt (Table S1). In terms of food-related anxiety, the question *“Do you ever feel anxious about eating certain foods due to fear of discomfort*, *such as choking or vomiting?”* was answered negatively by the majority (72%). However, 28% indicated they do feel such anxiety. A significantly higher proportion (88%) reported experiencing aversions to certain foods based on sensory characteristics such as smell or texture (Table S1). Women statistically significantly felt more anxious about consuming certain products, with the correlation coefficient being 0.189 (Table S2).

Additional chi-square analyses (Table S3) revealed that girls were significantly more likely than boys to engage in conscious weight control behaviors (χ^2^(2) = 11.09, *p* < 0.01) and to compare their bodies with others (χ^2^(2) = 8.49, *p* = 0.01). A statistically significant gender difference was also observed in breakfast timing (χ^2^(3) = 8.74, *p* = 0.03), while a trend was noted in emotional overeating (*p* = 0.06). Other comparisons, including place of residence, BMI category, physical activity, and calorie tracking, did not yield significant results. These behavioral tendencies were further reflected in the psychodietetic indicators summarized in Table S4. Girls exhibited slightly higher scores in emotional eating, knowledge–behavior gap, and media pressure indices, despite demonstrating marginally higher nutrition knowledge. Boys, in turn, reported slightly healthier behavior scores.

Supplementary Table S5 shows the distribution of nutrition knowledge levels and knowledge–behavior gaps by gender. The majority of adolescents fell into the “Moderate” knowledge category (68% of girls and 73% of boys), while “High” knowledge was more frequent among girls (17%) than boys (12%). Regarding the knowledge–behavior gap, 29% of girls were classified as having a “High” gap compared to 18% of boys, indicating that girls—despite having slightly higher nutrition knowledge—were less likely to translate this awareness into daily behavior. This reinforces the need to address psychological and contextual factors that may hinder behavior change, especially in female adolescents.

### 3.4. Adolescents and Awareness of the Basics of Healthy Nutrition

Due to the exploratory and cross-sectional design of this study, all reported associations are correlational and do not imply causality. In addition, the lack of control for confounders such as socioeconomic background, digital media exposure, and family dynamics further limits the interpretability of these findings.

Four questions in the survey were dedicated to assessing adolescents’ awareness of fundamental principles of healthy nutrition. The first question asked, *“Do you know what the Healthy Eating Plate is?”* More than half of the respondents (55%) reported familiarity with the concept, although this awareness was unevenly distributed—only 6% of male participants reported knowing about it, compared to 49% of female participants. The remaining respondents indicated they had not heard of the Healthy Eating Plate. Another question addressed knowledge of eating disorders and their underlying causes: *“Are you aware that eating disorders are characterized by persistent*, *abnormal eating behaviors such as food avoidance*, *selective eating*, *or binge eating*, *and that biological*, *social*, *and cultural factors influence these behaviors?”* An overwhelming majority of respondents (89%) demonstrated awareness of these defining features. Within this group, young women showed a greater level of awareness (64%) compared to their male peers.

The participants also demonstrated a good understanding of the term “satiety”. Over half (55%) accurately defined satiety as the feeling of complete hunger satisfaction, while 23% interpreted it as “eating until full”, and 22% as “absence of hunger”. Furthermore, the majority of adolescents correctly identified the normative range for BMI, with 71% providing the correct answer. The remaining participants selected incorrect responses (Table S1).

## 4. Discussion

Proper nutrition during adolescence plays a vital role in shaping long-term health outcomes. The period between ages 15 and 17 is particularly critical, as emotional and physiological changes can destabilize eating routines and contribute to the emergence of unhealthy dietary behaviors. Our study evaluated adolescents’ awareness of eating disorders and basic nutritional principles, revealing that although a majority of participants demonstrated theoretical knowledge, this awareness did not consistently translate into healthy daily practices. This gap has been highlighted in previous studies, which similarly reported that nutritional education alone does not always affect behavior change in youth [23,24].

Emotional dysregulation is increasingly recognized as a key transdiagnostic factor in the development of maladaptive behaviors, including disordered eating. This term refers to difficulties in managing and responding to emotional experiences in a flexible and adaptive manner. During adolescence, the prefrontal cortex—responsible for self-regulation and impulse control—is still developing, which contributes to higher emotional reactivity and susceptibility to using food as an emotional coping strategy [8,14,25]. Epidemiological data underscore the urgency of addressing eating disorders: in 2023, one in five teenagers reportedly struggled with disordered eating, and in 2019, approximately 3 million adolescents had a disturbed relationship with food due to body image concerns [25]. These trends are exacerbated by contemporary beauty standards and the normalization of restrictive diets, which significantly elevate the risk of developing eating disorders [2,13]. The increasing prevalence of overweight and elevated BMI among adolescents has also been associated with disordered eating behaviors [25].

A key area examined in our study was emotional eating. Approximately 39% of participants reported consuming high-calorie snacks in response to stress, aligning with findings by Guzek et al. [22] and Kotowska et al. [23], who observed that negative emotions often lead to the intake of ultra-processed foods. Emotional dysregulation, trauma, and psychosocial stressors—such as peer rejection or low socioeconomic status—have been linked to the onset of disorders like anorexia, bulimia, and binge eating [26,27,28]. These findings echo the conclusions of Olesen et al., who emphasized the role of contextual and familial stressors in compulsive eating patterns. From a neuropsychological perspective, the dual-process model may help explain these patterns. This model distinguishes between the impulsive system (fast, affect-driven) and the reflective system (slow, deliberate), with adolescents often relying more heavily on the former due to immaturity of cortical regions. This imbalance may lead to emotionally driven eating, particularly when combined with peer pressure and social comparison. Studies suggest that enhancing emotional awareness and regulation skills may buffer the effect of stress on disordered eating behaviors [10,11].

The COVID-19 pandemic further deteriorated the dietary choices of young people and amplified psychological vulnerabilities. Research shows that unhealthy dietary patterns formed during this period have persisted, with adolescents favoring processed foods rich in sugar and fat, which in turn negatively affect mood and body image [2,29]. Consequently, nutrition education programs should include components that promote healthy stress-coping strategies and emotional regulation, as emphasized by Katzman [30] and Sung et al. [31].

Our results also revealed distinct gender differences. Girls were more likely than boys to engage in binge eating during stressful periods, to experience guilt after consuming unhealthy food, and to restrict food due to body image concerns. These behaviors reflect heightened emotional sensitivity among adolescent girls, consistent with the findings of Skolmowska et al. [1]. These observations are supported by psychodietetic indicators calculated across the full sample (Table S4). Frequency distributions of nutrition knowledge levels and knowledge–behavior gap classifications by gender are summarized in Supplementary Table S5. Girls were more likely to fall into the “High Gap” category (29%) than boys (18%), further illustrating the disconnect between awareness and behavior in this group. Girls exhibited higher scores in emotional eating (EEI), media pressure, and knowledge–behavior gap indices, despite demonstrating slightly higher nutrition knowledge overall. These patterns suggest that psychosocial and emotional factors may hinder the application of nutritional knowledge in everyday behavior, particularly among female adolescents. Furthermore, girls were more influenced by appearance-related social media content, reinforcing the link between digital exposure and disordered eating. These observations are consistent with Park et al. [32] and Suhaga and Rauniyar [21], who reported that digital media significantly impact adolescents’ body satisfaction and can lead to technological addictions that disrupt healthy lifestyle choices.

The increasing use of platforms like TikTok, Instagram, and YouTube has enabled influencers to promote harmful behaviors, including restrictive dieting and the glorification of thinness. Some online communities support pro-anorexia narratives, normalizing dangerous practices such as laxative abuse or food avoidance [31,32,33,34,35,36,37,38]. Although there is ongoing debate about the extent of social media’s impact on eating disorders, numerous studies confirm that adolescents exposed to curated and idealized body images are more likely to develop dissatisfaction with their own appearance and engage in restrictive or compulsive eating behaviors [21].

Our findings showed that 47% of adolescents reported being influenced by social media in terms of body perception, with girls more strongly affected than boys. This supports previous studies by Wawrzyniak et al. [2] and Pelc [39], which identified appearance-related peer pressure as a powerful driver of emotional distress and maladaptive eating behaviors in girls. These effects are magnified by adolescents’ limited emotional maturity and their dependence on social acceptance during identity formation. As noted by Pelc [39], online shaming and exclusion may lead to symptoms of depression, anxiety, and disordered eating patterns.

Another notable observation is the widespread irregularity in meal consumption. A significant portion of adolescents (34%) reported eating breakfast more than two hours after waking, while 24% skipped it entirely. These habits can result in insulin resistance and increased risk of obesity, as confirmed by Tian et al. [40] and Schroder et al. [41], who also demonstrated that regular breakfast consumption improves cognitive performance. Consistent with our findings, previous research has linked meal skipping with low vegetable intake and poor food quality [41,42].

The traditional Polish diet, characterized by a high intake of flour-based products and sweets and a low intake of fresh vegetables, contributes to qualitative undernutrition among adolescents, despite the absence of calorie deficiencies [42]. Our study found that only 50% of respondents ate meals with their families daily and that most did not discuss nutrition at home. These observations echo those of Kotowska et al. [23], who emphasized the protective role of family mealtime and nutrition education in forming healthy eating habits.

BMI status also influenced adolescents’ behavior. Those with normal BMI were more likely to consume vegetables and maintain regular meal schedules, whereas overweight participants were more likely to snack and consume ultra-processed foods. Similar associations were reported by Mizia et al. [43] and Jeżewska-Zychowicz et al. [24], who linked early dietary patterns to long-term eating behaviors and weight regulation. Notably, over half of our respondents (52%) reported current or past attempts at weight reduction, with girls showing a much higher tendency to engage in dieting (63% vs. 24% for boys).

Dieting behavior in adolescence is often driven by body dissatisfaction and the internalization of unrealistic beauty standards. Prior experiences with parental weight control and critical comments about body shape may lead to disordered eating and reduced self-esteem in adolescence and adulthood [24,44]. Harmful behaviors such as fasting, vomiting, or excessive exercise are frequently used as coping strategies among adolescents, especially girls, to address body dissatisfaction [8,19,45].

Despite having basic knowledge about BMI, healthy eating principles, and satiety, many adolescents fail to apply this knowledge in practice. Our results align with those of Wawrzyniak et al. [2], who found a disconnect between theoretical awareness and actual dietary behavior. This emphasizes the need for holistic health education programs that address not only nutrition but also psychosocial factors such as peer pressure, emotional triggers, and media influence.

Interestingly, food selectivity related to smell or texture was reported by the majority of adolescents, indicating potential early symptoms of ARFID. Although most participants did not express strong food-related anxiety, nearly 88% declared an aversion to specific sensory characteristics. These results are in line with findings by Białek-Dratwa et al. [44], who called for increased awareness and education regarding ARFID in youth populations. While ARFID is more commonly diagnosed in younger children, our study supports the view that its features can emerge during adolescence and should be monitored in clinical and educational settings [45,46].

Our study also highlighted the relationship between physical activity and healthier food choices. Adolescents who reported regular physical activity were more likely to consume vegetables, lean proteins, and whole grains and to avoid processed foods. These findings are consistent with those of Liu et al. [47], who reported improved cognitive function and dietary quality among physically active youth.

Additionally, our study found that nutritional knowledge was higher among adolescents with overweight, possibly reflecting a greater personal concern with body weight. However, this awareness did not always translate into healthier behavior. This paradox mirrors observations by Całyniuk et al. [48], who noted that knowledge alone is insufficient when it is not supported by behavioral strategies or emotional regulation.

Finally, youth often rely on the internet as a primary source of health information, but this unfiltered content can expose them to extreme dietary trends such as “mukbang”. Research by Sung et al. [31] shows that watching such content is associated with higher intake of fast food, lower consumption of fruits and vegetables, and use of harmful weight control strategies. While our study did not explicitly assess media content preferences, the widespread normalization of binge-eating behaviors through online formats such as mukbang may influence adolescents’ perceptions of portion size and social eating, particularly among those already vulnerable to emotional dysregulation [29,49,50,51,52,53,54].

In summary, our findings reinforce the conclusions drawn in studies by Mizia et al. [43] and Tian et al. [40], highlighting the importance of integrated health education that includes nutritional, emotional, and digital literacy components. By addressing the psychosocial context of eating behavior, future interventions may more effectively support adolescents in forming lasting, healthy habits that persist into adulthood [55,56,57,58].

These findings underscore the importance of integrating emotional regulation strategies into school-based health promotion programs. These recommendations align with the transdiagnostic understanding of emotion dysregulation as a modifiable intervention target that may disrupt the progression from subclinical symptoms to full-threshold eating disorders. Psychological interventions that teach adolescents how to identify, tolerate, and manage emotional states, such as cognitive behavioral techniques or mindfulness-based stress reduction, could be crucial in preventing the progression from emotionally driven eating to clinically significant disorders. Targeting both nutritional knowledge and emotional skills in tandem may offer a more effective and developmentally appropriate approach to intervention. The distinction between knowledge, behavior, and emotional factors was quantitatively operationalized through psychodietetic indices and classification thresholds (Tables S4 and S5), enhancing interpretability beyond raw response frequencies.

## 5. Limitation Study

This study has several limitations that should be considered when interpreting the findings. First, the survey tool was custom designed by the authors, and although reviewed by experts, it was not psychometrically validated or piloted. This limits the generalizability and reliability of the constructs measured, particularly in domains such as emotional eating or body image perception.

Second, participants were recruited from a single secondary school in central Poland, which introduces potential selection bias and restricts the socio-demographic diversity of the sample. Moreover, the study focused exclusively on adolescents aged 15 to 17, despite the title referencing a broader age range (15–17). These age-related and regional limitations constrain the representativeness of the sample.

Third, the survey did not include variables such as socioeconomic status (SES), mental health diagnoses, parental education, or detailed digital media usage, all of which are known to affect dietary behaviors. Their omission prevented multivariate analyses or adequate control for confounding factors.

Fourth, the cross-sectional nature of the study precludes any inference of causality. All reported associations are correlational, and the lack of adjustments for multiple comparisons further limits the robustness of the statistical interpretations.

Lastly, the relatively small sample size (n = 120) reduces the statistical power to detect small effect sizes, and the gender imbalance (70% girls) may have influenced observed patterns in emotional responses and dietary behaviors.

## 6. Conclusions

This study highlights a persistent gap between nutritional knowledge and health-related behavior among Polish adolescents. While participants demonstrated awareness of concepts such as BMI, dietary guidelines, and the Healthy Eating Plate, their daily eating patterns often reflected irregular meals, emotional triggers, and social influences. Importantly, our findings support the role of emotional dysregulation as a mediator of unhealthy eating behaviors, particularly in adolescent girls. Social media exposure and peer comparison emerged as prominent psychosocial factors contributing to disordered eating tendencies. These insights suggest that future public health strategies should move beyond informational campaigns and incorporate psychological skill-building, particularly around emotional regulation and digital media literacy. Preventive interventions that integrate both cognitive and emotional components may more effectively reduce the risk of eating disorders and promote long-term health outcomes in adolescents.

## Figures and Tables

**Table 1:** The general characteristics of the studied population.

		Total	Young Woman	Young Man
Characteristics		*N* = 120	*N* = 87	*N* = 33
Age	arithmetic mean [years]	16.2	16.2	16.2
	variance [years]	0.5	0.5	0.5
	standard deviation [years]	0.7	0.7	0.7
	coefficient of variation [%]	4.4	4.5	4.3
Age distribution	15 years	20	15	5
	16 years	51	35	16
	17 years	49	37	12
Place of residence	village	45	32	13
	small town	37	28	9
	medium-sized town	33	25	8
	large city	5	2	3
Body mass index (kg/m^2^)	<18.0	26	21	5
	18.5–25.0	91	64	27
	>25.0	3	2	1

## Data Availability

All original data generated or analyzed during this study are available within the article and its Supplementary Materials.

## Supplementary Materials

The following supporting information can be downloaded at https://www.mdpi.com/article/10.3390/nu17121994/s1, Figure S1: Frequency of regular meals; Figure S2. Frequency of communal meals; Table S1. Questionnaire about healthy eating and lifestyle, risk of eating disorders, and knowledge about nutrition; Table S2. Association between qualitative variables and gender—statistical significance and strength of correlation (Cramér’s V); Table S3. Chi-square test results for gender comparisons; Table S4. Psychodietetic indicators by gender (n = 120). Table S5. Levels of Nutrition Knowledge and Knowledge–Behavior Gap by Gender (n = 120).
