# Supplementary material for: Awareness of Eating Disorders, Nutritional Knowledge, and Emotionally Driven Eating Among Polish Adolescents Aged 15–17—A Pilot Study
Source: Nutrients. 2025 Jun 13;17(12):1994. doi: 10.3390/nu17121994 (PMC12195971; doi:10.3390/nu17121994)
Supplement: Supplementary file 1 [file nutrients-17-01994-s001.zip › nutrients-3629226-supplementary.pdf]

nutrients

Article

Awareness of Eating Disorders, Nutritional Knowledge, and Emotionally Driven Eating Among Polish Adolescents Aged 15–17 – A Pilot Study

Marlena Zięba, Marta Jaskóła and Sabina Lachowicz-Wiśniewska*

[1] University of Kalisz (Calisia University), Faculty of Medicine and Health Science, plac Wojciecha Boguslawskiego 2 62-800 Kalisz, Poland, marlenaannazieba@gmail.com, m.jaskola@uniwersytetkaliski.edu.pl,
* corresponding author: s.lachowicz-wisniewska@uniwersytetkaliski.edu.pl

Abstract: Despite the growing awareness of nutrition and the popularity of healthy lifestyles among adolescents, disordered eating behaviors—such as anorexia nervosa, bulimia nervosa, binge eating disorder (BED), and avoidant/restrictive food intake disorder (ARFID)—remain significant public health concerns. ARFID, officially recognized only in 2013, is still poorly understood among youth. This study aimed to assess the relationship between adolescents' nutritional knowledge, emotional regulation, media influence, and eating behaviors. A cross-sectional study was conducted in 2024 among 120 students aged 15–17 attending W. Reymont Secondary School No. II in Ostrów Wielkopolski, Poland. Participants completed a custom-designed, paper-based questionnaire consisting of 30 single-choice questions and demographic items. The instrument assessed knowledge of eating disorders, body satisfaction, social media impact, and the emotional determinants of food choices. The tool was developed with expert input but has not undergone formal psychometric validation. While many adolescents demonstrated basic nutritional knowledge—such as awareness of BMI norms and food group distribution—they often failed to apply this knowledge to their dietary behaviors. Significant gender differences were observed: girls were more likely to restrict food intake, report emotional eating, and engage in slimming behaviors, while boys were less emotionally reactive and less influenced by social media. Most participants reported eating one meal daily with family but rarely discussed nutrition at home. Emotional involvement in eating, particularly among girls, emerged as a key factor, more influential than social media in shaping dietary behaviors. The findings highlight a clear gap between nutritional knowledge and actual behavior among adolescents, driven in part by emotional dysregulation and body image concerns. School-based interventions should incorporate not only nutritional education but also emotional regulation strategies and media literacy to effectively support healthy eating behaviors in youth.

Keywords: eating disorders; adolescent awareness; prevalence of disorders

Academic Editor: Firstname Lastname

Received: date
Revised: date
Accepted: date
Published: date

Citation: To be added by editorial staff during production.

Copyright: © 2025 by the authors. Submitted for possible open access publication under the terms and conditions of the Creative Commons Attribution (CC BY) license (https://creativecommons.org/licenses/by/4.0/).

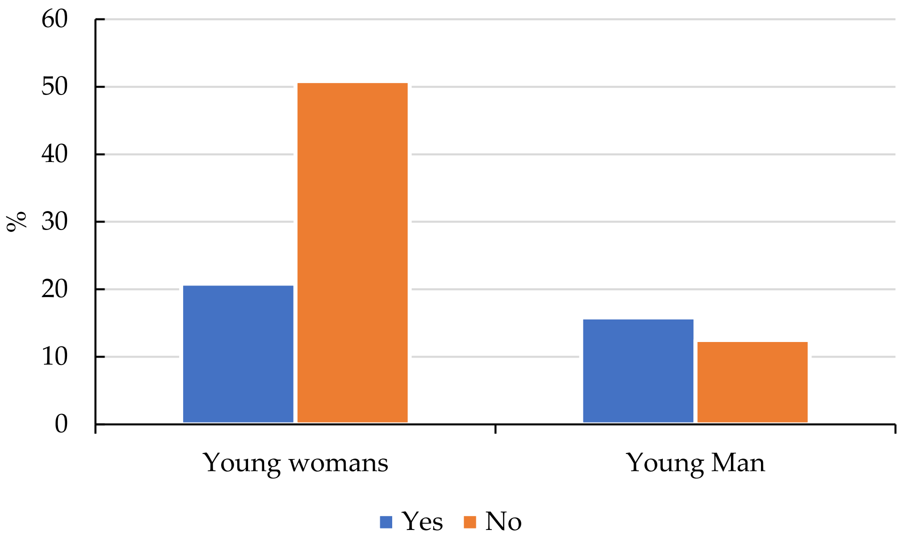

Figure S1. Declared importance of regular meals.

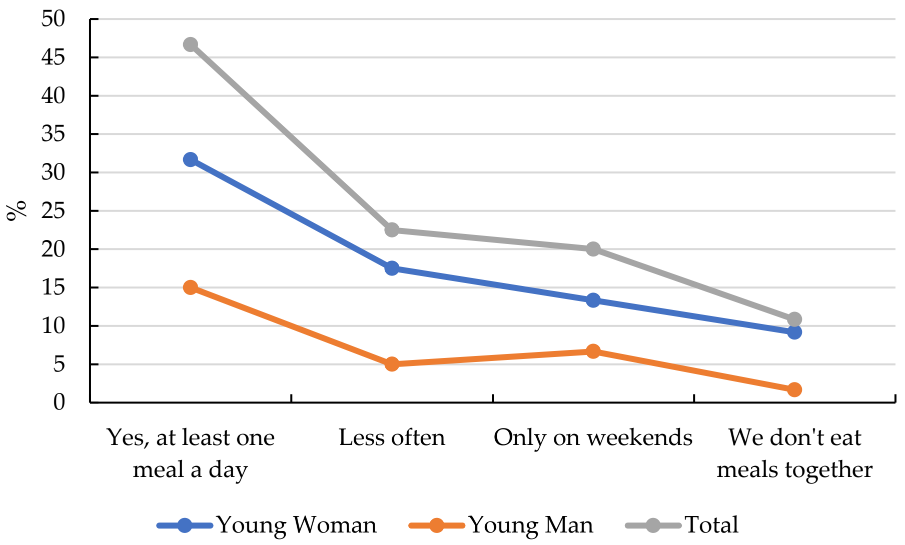

Figure S2. Declared consumption of communal meals.

Table S1. Questionnaire about healthy eating and lifestyle, risk of eating disorders, and knowledge about nutrition

**Characteristics**		**Young Woman**	**Young Man**	**Total**
		**N=87**	**N=33**	**N=120**
**Healthy eating and lifestyle**			**[%]**	
Do you deliberately skip meals?	Yes	23	4	28
	No	25	16	41
	Sometimes	23	8	32
Do you monitor your daily calorie intake?	Yes, I use a special app	8	1	8
	Yes, roughly „by eye"	20	9	29
	No, I don't count calories	44	18	63
Do you use stimulants (e.g., alcohol, cigaretts, others)?	Yes, occasionally	19	6	25
	Very often	5	3	8

	No, I don't use them	48	20	68
Do you add a portion of vegetables to main meals?	Always	22	6	28
	Sometimes	45	19	64
	I don't use them	5	3	8
Do you discuss healthy eating habits with your parents/guardians at home?	Yes	28	16	43
	No	44	13	57
**Risk of Eating Disorders**				
Do you eat food when experiencing high stress?	Rather yes	23	7	30
	Rather no	36	15	51
	Definiely yes	1	1	2
	Definiely no	12	6	18
Do you have binge eating episodes during sadness, doubt, boredom, or stress?	Sometimes	36	3	39
	Almost always	13	3	15
	Definietly not	23	23	46
Do you feel quilty after eating something unhealthy or in large quantities?	Sometimes	31	7	38
	Every time	12	1	13
	Definietly not	29	21	50
How often do you check your body weight?	Daily	5	4	9
	Once a week	18	4	22
	Once a month	18	7	25
	A few Times a year	22	12	33
	I don't check	9	2	11
Do you avoid certain foods due to fear of gaining weight?	Yes, I avoid such foods	26	3	29
	No, I eat everything in moderation	20	13	33
	I don't think about it	26	13	38
Do you feel anxious before eating ceratin foods due to the possibility of experiencing discomfort (e.g., choking, vomiting)?	No	48	23	72
	Yes, there are such foods	23	5	28
Are there certain smells or food textures that cause aversion (dislike)?	Yes, there are such foods	65	23	88
	No	7	5	12
Are you currently dieting, or have you ever been on a diet?	Yes	45	7	52
	No	27	22	48
Do you compare your body shape with others?	Yes, all the time	23	4	28
	Occasionally	36	11	47
	No	13	13	26
Do you feel stressed when eating in the presence of others?	No	35	26	61
	Yes, among peers	31	3	33
	Yes, with family	6	0	6
**Knowledge about nutrition**				
Do you know what the Healthy Eating Plate is?	Yes	49	6	55
	No	23	23	45
Do you know that eating disorders involve persistent abnormal eating behaviors,	Yes, I am aware	64	25	89
	I was not fully aware	8	3	11

including avoiding food, selective eating, binge eating, which are related to biological, social, and cultural factors?				
How do you define „satiety"?	Abstence of hunger	9	13	22
	Eating until completely full (stuffed)	19	4	23
	Feeling fully satisfied	43	12	55
Whas is the correct BMI range?	18,5 – 25	47	24	71
	Below 18,5	24	3	28
	Above 25	1	1	2

Table S2. Association between qualitative variables and gender – statistical significance and strength of correlation (Cramér's V)

Qualitative Variable 1	Qualitative Variable 2	Significance Level	Cramér's V	Remarks
Time of first meal:<br>- Immediately after waking<br>- Within 1 hour after waking<br>- More than 2 hours after waking<br>- I do not eat breakfast	Gender:<br>- Female<br>- Male	0.05	0.270	Females significantly more often skip breakfast. Males significantly more often consume breakfast immediately after waking.
Experiencing anxiety before consuming specific foods due to potential discomfort:<br>- No<br>- Yes, there are such foods	Gender:<br>- Female<br>- Male	0.05	0.189	Females significantly more often report anxiety before consuming specific foods.
History of weight-loss dieting (currently or in the past):<br>- Yes<br>- No	Gender:<br>- Female<br>- Male	0.05	0.322	Females much more frequently report having undertaken weight-loss dieting.
Comparison of own body shape with others:<br>- Yes<br>- Sometimes<br>- No	Gender:<br>- Female<br>- Male	0.05	0.266	Females significantly more often compare their body shape to that of others.
Avoidance of specific foods due to fear of gaining weight	Gender:<br>- Female<br>- Male	0.05	0.253	Females significantly more often avoid foods due to fear of weight gain, while nearly half of males do not consider this at all.
Binge eating episodes during sadness, doubt, boredom, or intense stress	Gender:<br>- Female<br>- Male	0.05	0.428	The vast majority of males do not experience binge eating episodes, while nearly half of the females do.
Intentional skipping of meals	Gender:<br>- Female	0.05	0.234	More than half of males do not skip meals intentionally, whereas most females do.

	- Male

Table S3. Chi-square test results for gender comparisons

Variable	*Chi²*	p-*value*
Age	0.67	0.715
Place of Residence	3.06	0.383
BMI	1.16	0.560
Breakfast Timing	8.74	0.033
Physical Activity	3.35	0.501
Emotional Overeating	3.45	0.063
Avoiding foods for weight reasons	2.32	0.508
Calorie tracking	3.05	0.549
Conscious weight control	11.09	0.0009
Body comparison with others	8.49	0.014
Perceived media influence	1.64	0.441

Table S4. Psychodietetic Indicators by Gender (n = 120)

**Gender**	**EEI Mean**	**EEI SD**	**Knowledge Mean**	**Knowledge SD**	**Behavior Mean**	**Behavior SD**	**Gap Mean**	**Gap SD**	**Media Index Mean**	**Media Index SD**	**N**
**Young Woman**	5.10	1.34	2.59	0.99	0.70	0.70	1.90	1.18	2.17	1.29	86
**Young Man**	5.12	0.98	2.29	1.03	0.88	0.81	1.41	1.16	1.59	1.40	34

Table S5. Levels of Nutrition Knowledge and Knowledge–Behavior Gap by Gender (n = 120)

Parameters	Level	Young Woman (N=87)	Young Man (N=33)
Knowledge	Low	12	6
	Moderate	59	24
	High	15	4
Gap	Low	12	8
	Moderate	49	20
	High	25	6
